# Feasibility intervention trial of two types of improved cookstoves in three resource-limited settings: study protocol for a randomized controlled trial

**DOI:** 10.1186/1745-6215-14-327

**Published:** 2013-10-10

**Authors:** Elizabeth Klasen, J Jaime Miranda, Subarna Khatry, Diana Menya, Robert H Gilman, James M Tielsch, Caitlin Kennedy, Robert Dreibelbis, Neha Naithani, Sylvester Kimaiyo, Marilu Chiang, E Jane Carter, Charles B Sherman, Patrick N Breysse, William Checkley

**Affiliations:** 1 Division of Pulmonary and Critical Care, School of Medicine, Johns Hopkins University, 1800 Orleans Ave, Suite 9121, Baltimore, MD 21205, USA; 2 CRONICAS Center of Excellence in Chronic Diseases, Universidad Peruana Cayetano Heredia, Lima, Peru; 3 Nepal Nutrition Intervention Project Sarlahi, Kathmandu, Nepal; 4 Department of International Health, Bloomberg School of Public Health, Johns Hopkins University, Baltimore, MD, USA; 5 School of Public Health, Moi University, Eldoret, Kenya; 6 Biomedical Research Unit, Asociacion Benefica PRISMA, Lima, Peru; 7 The Warren Alpert Medical School, Brown University, Providence, RI, USA; 8 Department of Environmental Health Sciences, Bloomberg School of Public Health, Johns Hopkins University, Baltimore, MD, USA

**Keywords:** Improved cookstove, Ventilated cookstove, Behavior change, Adoption, Indoor air pollution, DLCO, Spirometry, Biomass fuel

## Abstract

**Background:**

Exposure to biomass fuel smoke is one of the leading risk factors for disease burden worldwide. International campaigns are currently promoting the widespread adoption of improved cookstoves in resource-limited settings, yet little is known about the cultural and social barriers to successful improved cookstove adoption and how these barriers affect environmental exposures and health outcomes.

**Design:**

We plan to conduct a one-year crossover, feasibility intervention trial in three resource-limited settings (Kenya, Nepal and Peru). We will enroll 40 to 46 female primary cooks aged 20 to 49 years in each site (total 120 to 138).

**Methods:**

At baseline, we will collect information on sociodemographic characteristics and cooking practices, and measure respiratory health and blood pressure for all participating women. An initial observational period of four months while households use their traditional, open-fire design cookstoves will take place prior to randomization. All participants will then be randomized to receive one of two types of improved, ventilated cookstoves with a chimney: a commercially-constructed cookstove (Envirofit G3300/G3355) or a locally-constructed cookstove. After four months of observation, participants will crossover and receive the other improved cookstove design and be followed for another four months. During each of the three four-month study periods, we will collect monthly information on self-reported respiratory symptoms, cooking practices, compliance with cookstove use (intervention periods only), and measure peak expiratory flow, forced expiratory volume at 1 second, exhaled carbon monoxide and blood pressure. We will also measure pulmonary function testing in the women participants and 24-hour kitchen particulate matter and carbon monoxide levels at least once per period.

**Discussion:**

Findings from this study will help us better understand the behavioral, biological, and environmental changes that occur with a cookstove intervention. If this trial indicates that reducing indoor air pollution is feasible and effective in resource-limited settings like Peru, Kenya and Nepal, trials and programs to modify the open burning of biomass fuels by installation of low-cost ventilated cookstoves could significantly reduce the burden of illness and death worldwide.

**Trial registration:**

ClinicalTrials.gov NCT01686867

## Background

### Global burden of disease attributed to biomass fuel smoke

More than half of the world population burns solid fuels, including wood, dung, crop waste, coal and charcoal, indoors for cooking. Incomplete combustion of these materials results in the production of hazardous by-products that directly affect health [1,2]. The World Health Organization has identified indoor combustion of biomass solid fuels as one of the leading risk factors for disease burden worldwide. In 2010, more than 3.5 million deaths and 4.3% (108 million) of global disability-adjusted life years (DALYs) were attributed to biomass fuel smoke [3]. Biomass fuel smoke appears to affect all household members adversely [4], women and children tend to have the highest risk of exposure to high levels of indoor air pollution due to their involvement in the cooking process. In developing countries, biomass fuel exposure has been associated with an increased risk of respiratory infections including pneumonia [5], lung cancer [6,7], chronic obstructive pulmonary disease (COPD) [8-17], cardiovascular events, low birth weight and all-cause mortality in both adults and children [18].

### Biomass fuel exposure as a risk factor for chronic respiratory disease

COPD is one of the most important causes of morbidity and mortality in people 40 years of age and older, and it is a disease with a substantial and increasing socioeconomic burden [19,20]. A large-scale, population-based study conducted in five Colombian cities [4] found that cooking for 10 years or more with a wood stove was an independent risk factor for COPD after adjustment for multiple confounders (odds ratio = 1.5, 95% CI 1.4, 2.4). The burden of COPD deaths is also disproportionally concentrated in low- and middle-income countries (LMIC), which accounted for nearly 90% of global deaths from COPD in 2001 [21]. In 2010, COPD ranked third in causes of morbidity worldwide and was responsible for 2.9 million deaths [22] and 4.7% of global DALYs lost [23]. By 2020 it is projected that COPD will be the fourth leading cause of DALYs lost in LMIC [24]. In China alone, for example, COPD is expected to kill 65 million people between 2003 and 2033 [25], and this number is expected to increase with an aging population [26,27]. Approximately 50% of deaths from COPD in LMICs are thought to be attributable to the open burning of biomass fuels, and it is estimated that 75% of these deaths occur in women [1].

### Respiratory outcomes that may change acutely after exposure to biomass fuel smoke

Studies of the natural history of COPD usually require large numbers of patients and long periods of follow up to detect differences between interventions on incident rates of COPD. In studies of shorter duration, the forced expiratory volume at one second (FEV_1_) and the rate of decline in FEV_1_ are the most widely used outcome measurements in short observational studies and clinical trials of COPD [28]. FEV_1_ is central to the definition of COPD and classification of its severity [28]. However, longitudinal studies that focus only on the rate of FEV_1_ decline in response to an intervention may also require several years to manifest or detect in a population-based study. Another respiratory outcome that may change more acutely after the installation of an improved cookstove is peak expiratory flow (PEF). PEF is defined as the highest flow achieved at the mouth during a forced vital capacity maneuver [29]. PEF can be easily measured with a spirometer or peak-flow meter and is commonly used in environmental epidemiology studies to monitor acute changes in lung function after occupational or toxicological exposures [30-32]. Another measure that would be expected to change rapidly with successful implementation of an improved cookstove is carboxyhemoglobin [33], which can be easily measured with non-invasive techniques. A direct measure of carbon monoxide (CO) exposure is exhaled CO measured in parts per million. Exhaled CO levels rise and fall dependent on the participant’s exposure to CO in the preceding 2 to 3 days and therefore can accurately classify present exposure levels.

An important measure of lung function is diffusing capacity of the lung for CO (DLCO). Measurement of the DLCO provides information on pulmonary gas transfer. Abnormalities in diffusing capacity of the lung are probably one of the earliest indications of emphysematous changes due to cigarette smoke or biomass fuel exposure, and a significant decline in DLCO may even precede the development of obstruction. However, there is limited work on early changes in DLCO in response to biomass fuel exposure. A challenge in measuring DLCO in field studies has been the portability of testing equipment and the availability of gas mixture; however, new developments in technology have made this equipment more portable and accessible. The current study will provide us with an opportunity to pioneer the measurement of DLCO in field studies, understand the effects of chronic exposure to biomass fuels on DLCO variability, and to determine if there are changes in DLCO after intervention with an improved, ventilated cookstove.

### Previous research on improved, ventilated cookstoves

Improved, ventilated cookstoves have been introduced as a potential method to considerably reduce exposure to indoor biomass fuel smoke [34]. However, in field trials where reductions in environmental emissions were achieved with improved cookstoves, a concomitant effect on health outcomes has not always been evident. For example, a large, randomized field trial of improved cookstoves in Guatemala was unable to measure an important reduction in physician-diagnosed childhood pneumonia despite a reduction of 50% in personal environmental emissions after intervention with an improved chimney stove [35]. The authors attributed a lack of an effect due to an insufficient exposure reduction; however, they did not expand on reasons why their intervention failed to achieve important reductions in environmental emissions. Similar reductions in indoor air pollution of 40 to 60% with improved cookstove interventions have been reported in Peru, China, and India [36-38].

Little is known about how the determinants of improved cookstove adoption and sustained use, the role of individual perceptions of improved cookstoves as they are shaped and the perceived benefits and limitations of specific cookstove designs affect environmental exposures and health outcomes. The difficulty of improved cookstove interventions is in eliciting a two-fold behavioral change by the user to cease in the use of the traditional stove and to uptake the new technology [39]. Mixed-methods approaches are thus needed to understand the behavioral, cultural and social barriers to successful improved cookstove adoption and for accurately determining the potential of improved, ventilated cookstoves as effective methods for removing biomass fuel smoke and removing the burden of chronic respiratory disease in LMICs [39-41]. We aim to fill this gap in knowledge by conducting a comprehensive study that encompasses evaluation of environmental, respiratory and cardiovascular health outcomes, and behavioral changes before and after an intervention with improved cookstoves with a chimney vent to the exterior, and analyze the influences on the primary cookstove user that determine her preferences for specific stove designs, motivators and barriers to adoption, and contextual data on the key behaviors associated with cooking.

## Methods

### Study objectives

This study is a multi-country, feasibility intervention trial of improved, ventilated cookstoves to inform a future, large-scale field trial. We aim to characterize environmental, health and respiratory outcomes in three diverse resource-limited settings (Kenya, Nepal and Peru) with the goal of providing a more robust understanding of the improved cookstove adoption process and factors related to sustained use of improved, ventilated cookstoves. We will install two different types of improved cookstoves with a chimney, in rural households where biomass fuels are used almost exclusively for cooking, and evaluate the intervention based on the following specific aims:

1. Compare particulate matter (PM) and CO concentrations between traditional cookstoves (before intervention) and two types of improved cookstoves with a chimney (after intervention).

2. Compare respiratory outcomes (spirometry, PEF, exhaled CO and DLCO) in women aged 20 to 49 years between traditional cookstoves (before intervention) and the improved cookstoves with a chimney (after intervention).

3. Assess behaviors, preferences and attitudes that shape improved cookstove adoption and intervention compliance among users of three types of cookstoves; evaluate the reasons for their preferred choice of cookstove; and, assess the potential for sustained maintenance and use of the intervention.

A secondary, health outcome aim is to compare blood pressure in women aged 20 to 49 years between traditional cookstoves (before intervention) and the improved cookstoves with a chimney (after intervention).

### Study design

**Figure 1:**
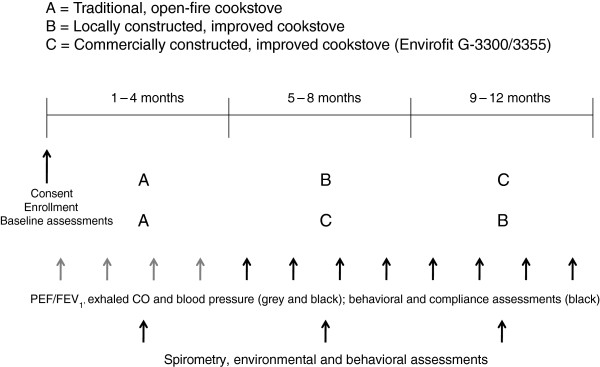
**Intervention study design by site.** PEF = peak expiratory flow, FEV_1_ = forced expiratory volume at 1 second, CO = carbon monoxide.

**Figure 2:**
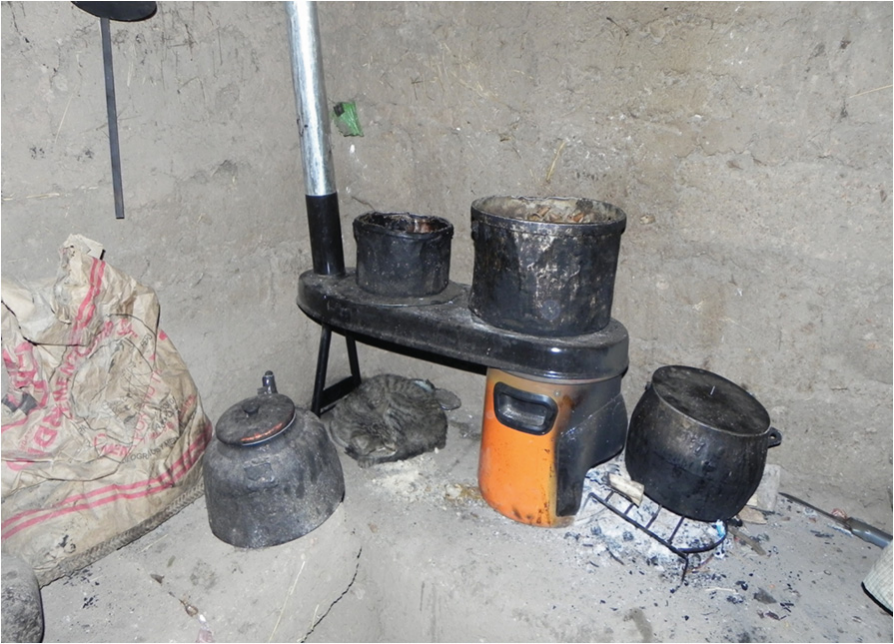
**Envirofit G**-**3300**/**3355 installed in Peru.**

**Figure 3:**
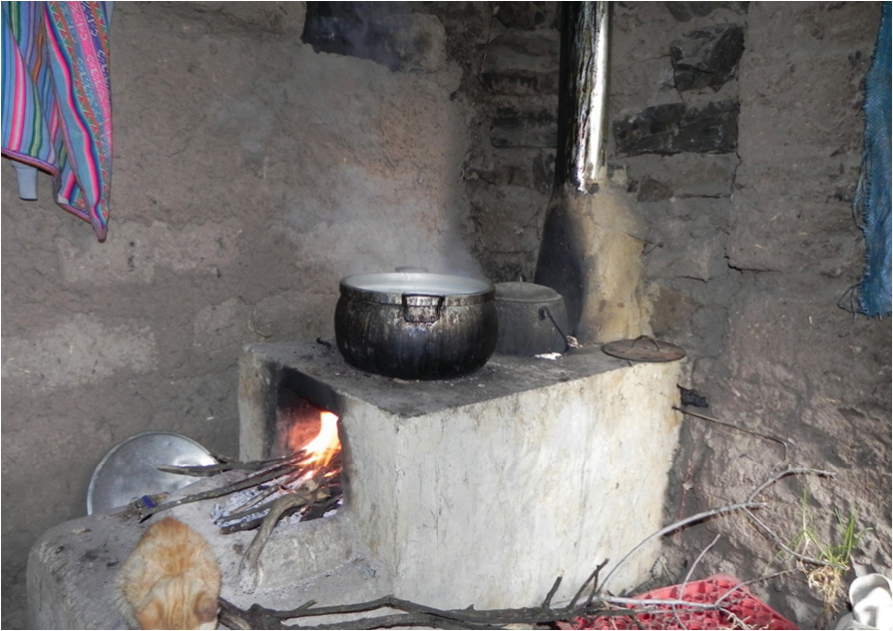
Locally constructed, improved cookstove installed in Peru.

**Figure 4:**
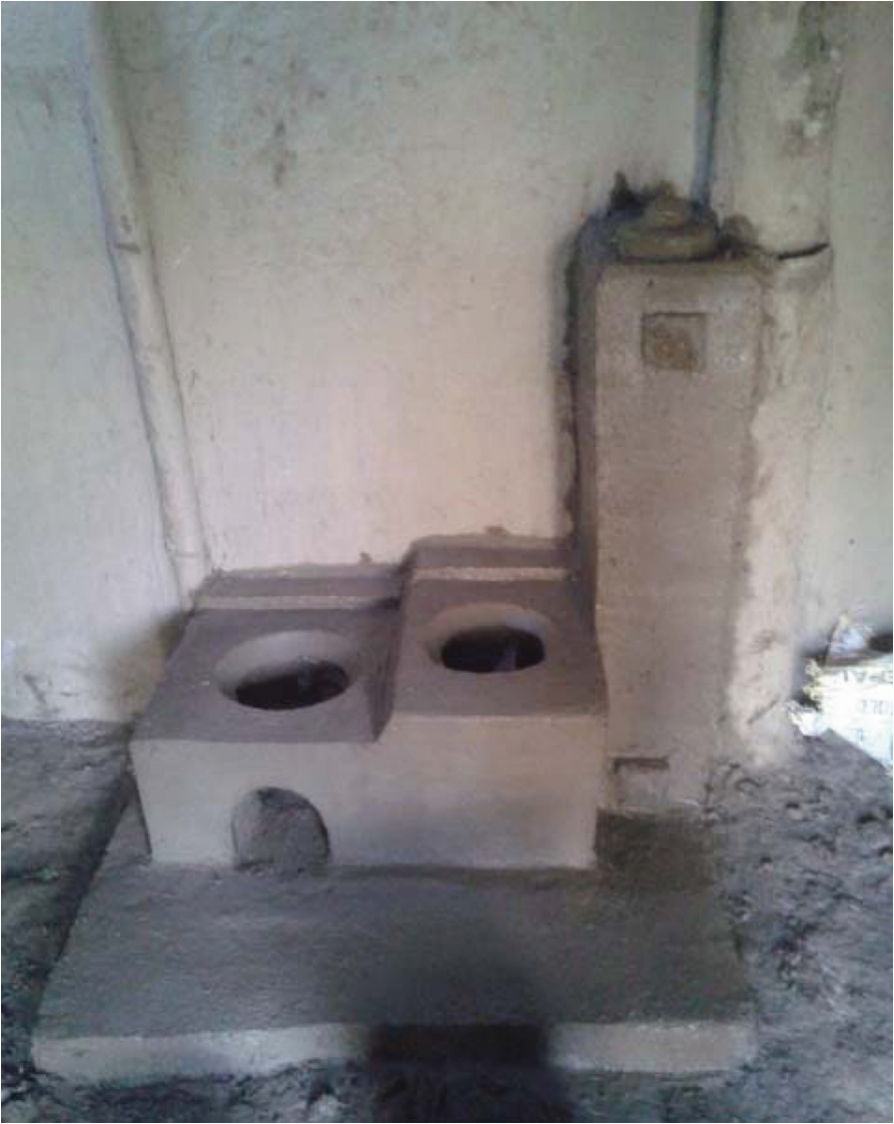
Locally constructed, improved cookstove installed in Nepal.

**Figure 5:**
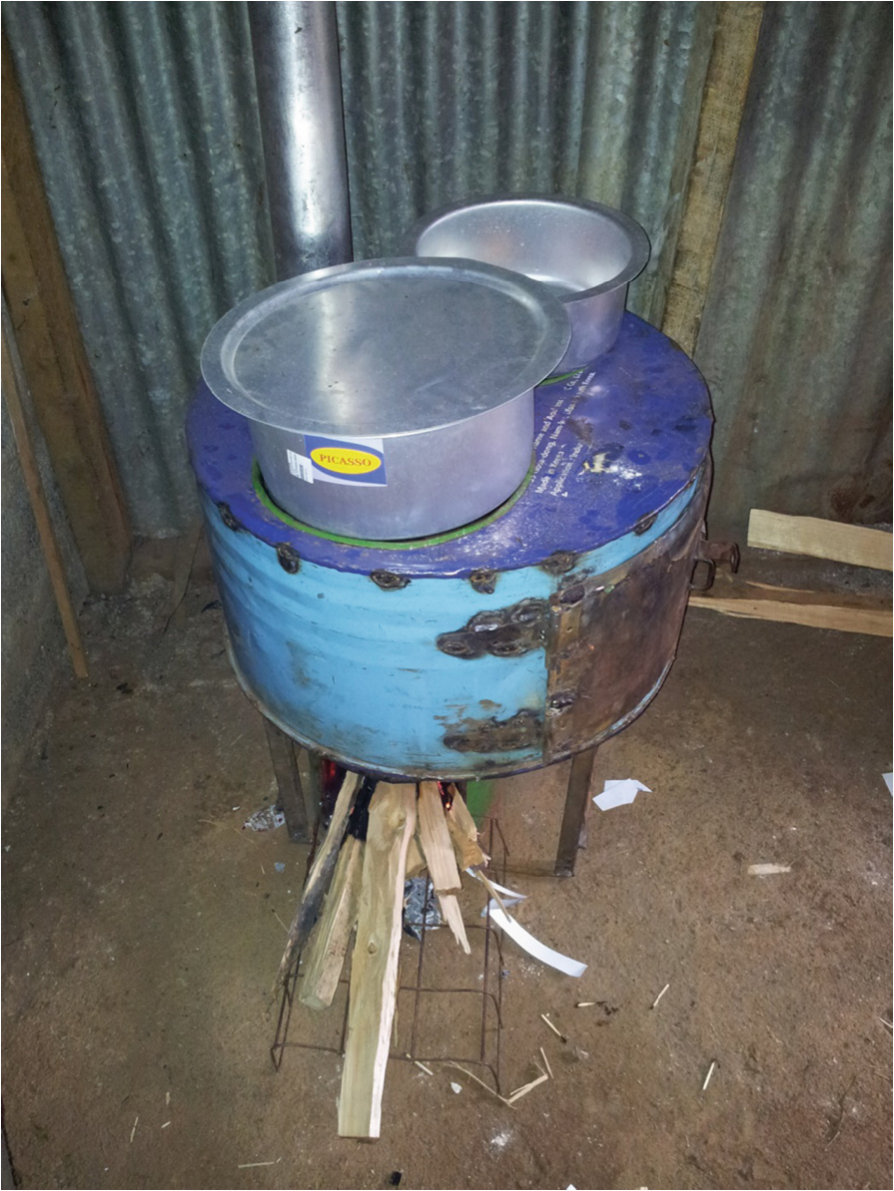
Locally constructed, improved cookstove installed in Kenya.

Our study design will be a randomized crossover intervention trial in which all participants will first have a run-in observational period of four months with their traditional cookstove prior to randomization into two intervention arms (Figure 1). At baseline, we will obtain sociodemographic information for each household, respiratory outcomes and other health data for the participating women. Qualitative methods, such as interviews and direct observations, and quantitative questionnaires will be used during the observational period to collect data on fuel use, the cooking process, and the participant’s current perception of their traditional cookstove. One of the intervention arms will first have the commercially constructed, ventilated cookstove installed in their kitchen (Figure 2). They will then be followed for four months. At crossover, we will install a locally constructed, ventilated cookstove and follow the participants for another four months. The other intervention arm will first receive the locally constructed cookstove at the beginning of the first four-month intervention period followed by installation of the commercially constructed improved, ventilated cookstove for the second four-month intervention period. The materials and method of construction of the locally constructed cookstove will vary by site (Figures 3, 4, 5).

During each of the two four-month intervention periods, we will request that the woman participant exclusively uses the improved, ventilated cookstove installed for that period. We will assess the intervention adoption process, compliance with cookstove use, respiratory health and blood pressure monthly. Environmental measures will be taken at least once at baseline and during each intervention period. Spirometry will be measured once during the baseline period and at the end of the intervention periods. DLCO will be measured once during the baseline period and at the end of the intervention periods in Peru only. Participants will participate in in-depth interviews every period discussing their opinions of the current stove they are using and previous stoves used. The cooking process will be directly observed at least once per period to record changes in cooking-related behaviors and practices. At the end of the second four-month period, we will crossover and install the second type of improved, ventilated cookstove and follow all participants for another four months. Following each four-month period a focus group will be conducted with study staff. The study will require 12 consecutive months to complete at each site.

### Study organization

The trial will be implemented at the United States National Heart, Lung and Blood Institutes and UnitedHealth Group Centers of Excellence in Chronic Diseases in Kenya (ASANTE Center of Excellence) and Peru (CRONICAS Center of Excellence) and at the Nepal Nutrition Intervention Project, Sarlahi (NNIPS) in Nepal. Partners include Johns Hopkins University in Baltimore, USA and Brown University in Providence, USA. All three field sites have local offices and staff that provide sufficient and culturally sensitive infrastructure for recruitment, intervention, data collection and data entry for the implementation of this project. This study was approved by the Institutional Review Boards of Universidad Peruana Cayetano Heredia (Lima, Peru), Johns Hopkins Bloomberg School of Public Health (Baltimore, USA), A.B. PRISMA (Lima, Peru), Moi University (Eldoret, Kenya), Institute of Medicine at Tribhuvan University (Kathmandu, Nepal) and Lifespan/The Miriam Hospital (Providence, USA).

### Study setting and populations

The study will take place in three rural, resource-limited settings: the Ndanai sub-location of the Uasin Gishu district in western Kenya, the southern district of Sarlahi in Nepal, and the Vinchos district of Ayacucho in the foothills of the Andes in Peru. Households in the three study settings use biomass fuels almost exclusively as the primary source of energy for cooking and heating. The site in Kenya is located in a rural setting in Rift Valley, Kenya at approximately 2,200 meters above sea level. Wood and crop residues are the main sources of biomass fuel for this population. The site in Nepal is located in the low-lying, southern plains of the Sarlahi District (the Terai) at 300 meters above sea level. The study area encompasses 30 Village Development Committees with a total population of approximately 190,000 inhabitants. More than 95% of the population under study burn biomass fuels and primary sources of biomass fuel include wood, dung and crop waste. Its culture and geography are similar to other areas of southern Nepal, northern India and Pakistan, and western Bangladesh. The site in Ayacucho, Peru is located in the Andes at altitudes ranging between 3,000 and 4,000 meters above sea level. Ayacucho is among the poorest departments in Peru, with an extreme poverty rate of 45%, an illiteracy rate of 43% among women. Among the poor populations, 75% live in adobe huts and 91% burn biomass fuels. Wood and dung make up the majority of the biomass fuel burned by the population.

Each site will enroll 40 to 46 women for a total study population of 120 to 138 participants. To be eligible for enrollment, the woman must meet the following criteria: be between 20 and 49 years of age; be the main household cook; primarily use a traditional, open-fire cookstove indoors in a structure with at least three walls and a roof; be willing to have two types of improved cookstoves installed and agree to use them during the intervention period to which they are assigned; have household walls of mud, brick, cement, or wood; be a full-time resident of the intervention community capable of providing informed consent; and participate in all study procedures.

### Intervention

The two types of improved, ventilated cookstove incorporate the following characteristics: at least two burner openings; a cleanable entrance and combustion chamber for biomass fuel; a flue designed to draw air into the fire box and pass it out through a chimney; a chimney, vented to the exterior, of adequate height to create a draft; an exterior port designed to prevent the backflow of smoke and an access port for the chimney that permits cleaning. There are multiple commercially constructed and locally constructed models available with the above specifications. To assess cookstove functionality over time and the multifaceted determinants of use involved with an improved, ventilated cookstove intervention we will install and compare both a commercially-available and a locally-constructed cookstove in each enrolled household.

We selected the Envirofit G-3300/3355 (http://www.envirofit.org) for installation in every site and a site-specific locally-constructed improved, ventilated cookstove for use in the trial. We piloted multiple locally-constructed improved, ventilated cookstoves. The goal of this pilot study was to compare ability to reduce indoor air pollution, the reliability between cookstoves of the same type, the functionality over time, preferences and cultural compatibility, and cost.

The installation and explanation of the cookstoves will be important for their acceptance and sustained maintenance and use. During installation, complete household assistance and observation during the process will be encouraged. Education on the intervention will be mandatory for participating women, although all family members will be encouraged to attend.

### Sample size

Since this study was designed as a feasibility trial to inform a future, large-scale field trial, we did not calculate a sample size to demonstrate an intervention effect for our primary outcomes. However, the study sample should provide a sufficient indication of the relationship between decreased indoor biomass fuel smoke exposure and improved respiratory health outcomes across and among interventions with improved cookstoves. In addition, the sample size allows for the combination of qualitative and quantitative data collection and monthly respiratory and compliance follow-up.

### Baseline assessments

We will collect the following sociodemographic data at enrollment: household demographics, including the age and sex of all persons; household construction including the materials used for the walls, roof, and floor, number of rooms and floors; household socioeconomic status; information on cooking practices and types of fuels used with traditional cookstoves; and exposure to tobacco smoke (number of persons smoking in the home and amount).

We will also collect baseline data on respiratory outcomes using a modified version of the study questionnaire from the Burden of Obstructive Lung Disease (BOLD) study [26]. The baseline data will also include anthropometric measurements (height and weight), total hemoglobin (g/dL) measured non-invasively by fingertip arterial blood sensor (Masimo Pronto or Pronto 7, Irvine, CA, USA), a measurement of exhaled CO and blood pressure. We will include a detailed questionnaire about personal history of smoking and second-hand smoke.

### In-depth interviews

In-depth interviews will begin during the traditional cookstove observation period and continue through both intervention periods. Their purpose is to collect information on how participants view their current cookstove, perceptions of indoor air quality, and the social and cultural context in which cooking and stove preference and use is embedded. All interviews will be led by the participant with the interviewer guiding the direction of the interview as they probe for relevant information based on an interview guide. Interviews will occur at least once per period in a setting of the respondent’s choice, generally in the household. Interviews will be conducted in the local language and are expected to last between fifteen minutes and one hour.

### Semi-structured interviews

Semi-structured interviews will be carried out monthly throughout the intervention periods to closely follow the adoption process as participants transition from one type of improved, ventilated cookstove to the other, and understand related opinions and behaviors. Questions will be less open-ended than those in the in-depth interviews and responses are expected to be shorter and more concise.

### Observations

Structured observations will be used to determine typical cooking times and lengths, type of foods cooked and individuals present during stove use. During the observation, a staff member will sit in an unobtrusive place in the household in sight of the main cooking practices and observe use and handling of existing stoves, types of food prepared, and how cookstoves affect food preparation and dynamics within the household, while taking notes. Observations will be conducted either during morning or evening cooking practices and are expected to last from meal preparation to cooking completion. Staff members will be instructed not to interact with the participants except as necessary for politeness. All household members present at the time of the observation will be asked if they are comfortable being present during the observation period, and if any household member does not agree to observation, cooking practices in that household will not be observed at that time.

### Focus groups

Focus groups will be conducted with data collection staff. Their perspectives and experiences with both traditional and improved cookstoves in the course of trial implementation will complement the perspectives of participants gained from interviews. Focus groups will occur following each study period of four months and will be limited to individuals of the same general rank within the project to avoid having supervisors and their staff in the same discussion. Focus group participants will be informed that their participation in the focus group will not affect their employment with the study, and they are free to decline to answer any question they feel uncomfortable answering.

### Longitudinal behavioral and compliance data

We will collect both quantitative and qualitative behavioral data and cookstove use compliance data monthly during the study periods. Compliance with improved cookstove use will be assessed by asking women how many total meals were cooked per day in the household in the preceding two weeks and how many were cooked using the new cookstove. We will ask how often a cookstove was used for a purpose other than cooking, what proportion of those times the cookstove was used, and what types of fuel sources were used. We will also determine if the intervention cookstove was not used during its corresponding intervention period and the reasons for the decision. Any modifications to the new cookstoves made by the families and any damage or breakdown of the cookstove will also be recorded during these monthly visits. We will also ask about length of cooking time to better understand whether the new stoves alter cooking time, which could be important for understanding compliance and acceptance.

In addition to the structured survey data on compliance and practices, we will use semi-structured interviewing techniques to assess individual preference and attitudes toward cookstoves and ways in which participants have adapted existing cooking practices and stove-use practices. Longitudinal qualitative interviews will provide an opportunity to assess how individual attitudes towards cookstoves, and ultimately acceptance of the new technology, are shaped over time and with repeated use. We will also assess changes in cooking practices over time. At the end of the trial, we will ask participants to indicate their preferred cookstove and to complete a final in-depth interview regarding individual preference, experiences using all three cookstove varieties, and the perceived benefits and drawbacks of each stove.

### Environmental assessment

To assess indoor air quality, we will measure 24-hour indoor air PM and CO concentrations at least once during the baseline period and again during each intervention period. Before the start of every study period, all environmental sampling equipment will undergo quality control checks according to manufacturer instructions to ensure quality performance across all machines. We plan to measure particulate matter with the pDR-1000 (Thermo Scientific, Franklin, MA, USA) passive sampler for a 24-hour period. This device incorporates a pulsed, high output, near-infrared light-emitting diode source. The intensity of the light scattered over the forward angle by particles passing through the sensing chamber is linearly proportional to airborne PM concentration. The pDR-1000 detects particles of 0.3 to 2.0 μm in size more efficiently than 2.0 to 10.0 μm [42-44]; however, there is a high level of agreement between PM determined by the pDR-1000 and gravimetric measurements of PM_2.5_[42,44]. Therefore, PM measured with the pDR-1000 is a good approximation for PM_2.5_. Every machine will be calibrated to zero-air before deployment in the field. We will measure relative humidity (RH) using the HOBO Data Logger (Onset Corp., Bourne, MA, USA) to adjust PM by RH [45]. We will carry out the measurements of ambient CO in the same 24-hour period as the PM measurements with the EasyLog USB CO Monitor (Lascar Electronics, Eerie, PA, USA). We will place the environmental measurement setup approximately 1.0 to 1.5 m above the floor and <1.0 m away from the current cookstove.

### Health outcomes

We will measure height and weight once at baseline for each individual using standard techniques [46]. The following will be measured, beginning at baseline, monthly throughout the trial:

• PEF and FEV_1_: the Piko-1 (nSpire Health, Inc., Longmont, CO, USA) will electronically measure PEF and FEV_1_. We will obtain at least two PEF measurements within 70 L/minute and at least two FEV_1_ measurements within 0.90 L.

• Exhaled CO levels and carboxyhemoglobin (% COHb): a Micro CO (Micro Direct, Inc., Lewiston, ME, USA) will electronically measure exhaled CO (ppm) and % COHb. Any exhaled CO measurement over 10 ppm will be repeated up to three times until two measurements are obtained within ± 2 ppm. The average of the obtained measurements will be reported.

• Pulse oximetry: arterial saturation of oxygen, perfusion index and pulse will be measured by fingertip sensor with a Masimo Rad 5v (Masimo, Irvine, CA, USA).

• Blood pressure: systolic and diastolic blood pressure will be recorded with Omron Healthcare 10 Series (Omron Healthcare, Inc., Lake Forest, IL, USA). The device has an internal calibration check system.

We will measure the following respiratory outcomes at least once at the mid-point of each four month period:

• Spirometry: we will use a flow-based spirometer (EasyOne Pro, ndd Zurich, Switzerland) to measure lung function. We will record forced vital capacity (FVC), FEV_1_, FEV_1_/FVC, PEF and flow-volume curves. We will obtain at least three acceptable and reproducible spirometric maneuvers in accordance with the joint European Respiratory Society and American Thoracic Society guidelines [47]. A field supervisor will review flow-volume curves with technicians on a daily basis to evaluate for quality. De-identified flow-volume curves will be transmitted on a weekly basis to Baltimore for additional over-read and grading. 

• DLCO: this measurement will only be conducted in Peru. We plan to measure DLCO as it is feasible using the EasyOne Pro (ndd, Zurich, Switzerland). We will follow standard criteria from the American Thoracic Society for the measurement of DLCO [48], and adjust values of DLCO for altitude, hemoglobin and COHb levels.

### Data management

The system for data collection in the field will be primarily paper based. For the social and behavioral component, we will collect detailed field notes from each household visit and audio-recorded interviews that will be transcribed by local field staff and translated into English. Data processing and storage will be centralized at the Data Coordinating Center in Peru. Data will be stored at a central server in the Data Coordinating Center and will be shared with all collaborators.

## Discussion

We plan to perform a one-year, community-based feasibility trial in which we will install two types of improved cookstoves with a chimney, in 40 to 46 rural households of women aged 20 to 49 years, in three resource-limited settings. Improved cookstoves have been investigated as a solution to prevent or reduce lung disease in low- and middle-income countries, especially in women and children. However, previous research on improved cookstoves has focused on exposure, fuel consumption and health outcomes. To date, limited data exists on the social, economic, and behavioral influences on successful cookstove integration measured by exposure reduction and positive health outcomes. As preferences for specific stove designs, attitudes towards using new technologies, and contextual data on the behaviors associated with cooking may also vary by cultural context, a better understanding of how use is adapted over time, how individual perceptions of improved cookstoves are shaped, and perceived benefits and limitations of improved cookstoves is needed [49]. If this trial demonstrates that reducing indoor air pollution is feasible and potentially effective in resource-poor settings like Peru, Kenya and Nepal, programs designed to modify the open burning of biomass fuels by installation of low-cost, ventilated cookstoves could significantly reduce the burden of COPD illness and death.

Our study has some potential shortcomings. First, the overall number of participants enrolled in this trial is small. As the trial was designed as a feasibility study spanning three countries with a longitudinal qualitative component, the number of participants able to be enrolled was restricted. However, as designed, the study participants will serve as their own controls and we will be able to evaluate their experiences with a locally constructed, ventilated cookstove and a commercially constructed, ventilated cookstove. Second, we may incur bias in self-reported symptoms after installation of the intervention due to participant awareness of the health benefits associated with improved cookstoves. Another potential limitation of the study is not testing a movement up the fuel ladder to a cleaner fuel, such as liquid-propane gas, biogas or electric cookstoves. However, a cleaner fuel is not an appropriate intervention for our intended populations at this time, as the selected rural populations do not have the supply system or financial ability to sustain an intervention aimed at upward movement on the fuel ladder. Cleaner fuels are the way of the future but to address the burden now, we must focus on appropriate methodologies that utilize currently available energy sources [50].

## Trial status

Institutional Review Board approval has been secured for all sites and the trial has been registered with http://www.clinicaltrials.gov. Data collection has begun at all sites.

## Abbreviations

CO: Carbon monoxide; COHb: Carboxyhemoglobin; COPD: Chronic obstructive pulmonary disease; DALY: Disability-adjusted life year; DLCO: Diffusing capacity of the lung for carbon monoxide; FEV1: Forced expiratory volume at one second; FVC: Forced vital capacity; LMIC: Low- and middle-income countries; PEF: Peak expiratory flow; PM: Particulate matter; RH: Relative humidity.

## Competing interests

The authors declare that they have no competing interests.

## Authors’ contributions

WC, JM, SKh and DM conceived the original study design. EK, MC, RG and WC were responsible for conduct of the study in Peru; NN, SKh and JT were responsible for conduct of the study in Nepal; and DM, SKi, JC and CS were responsible for conduct of the study in Kenya. PB provided expert guidance in the design and conduct of the environmental exposure assessment. CK and RD provided expert guidance in the design and conduct of social behavioral assessment. EK and WC led the writing of the manuscript. WC had ultimate oversight over the study design and conduct of this trial. All authors contributed equally to the development of the study design, and conduct, and the writing of the manuscript. All authors read and approved the final manuscript.

## Authors’ information

Members of the COCINAS Trial Working Group: Angela Huaman (A.B. PRISMA), Mariela Levano (A.B. PRISMA), Delia Haustein (A.B. PRISMA), Evelyn Rhodes (Johns Hopkins University), Laura Grajeda (Johns Hopkins University), Stephanie Levy (Johns Hopkins University), Steven LeClerq (Johns Hopkins University), Robert Wise (Johns Hopkins University), Priscah Mosol (Moi University), Francis Ogaro (Moi University), Cosmas Apaka (Moi University), Joyce Baliddawa (Moi University).
